# Inactivation of Semicarbazide-Sensitive Amine Oxidase Stabilizes the Established Atherosclerotic Lesions via Inducing the Phenotypic Switch of Smooth Muscle Cells

**DOI:** 10.1371/journal.pone.0152758

**Published:** 2016-04-04

**Authors:** Ya Peng, Jun Wang, Miao Zhang, Panpan Niu, Mengya Yang, Yilin Yang, Ying Zhao

**Affiliations:** 1 Department of Neurosurgery, The First People’s Hospital of Changzhou, Soochow University, Changzhou, 213003, China; 2 Institutes of Biology and Medical Sciences, Soochow University, Suzhou, 215123, China; 3 Department of Pathophysiology, Soochow University, Suzhou, 215123, China; 4 Modern Medical Research Center, The First People’s Hospital of Changzhou, Soochow University, Changzhou, 213003, China; Monash University, AUSTRALIA

## Abstract

Given that the elevated serum semicarbazide-sensitive amine oxidase (SSAO) activity is associated with the severity of carotid atherosclerosis in clinic, the current study aims to investigate whether SSAO inactivation by semicarbazide is beneficial for established atherosclerotic lesions in LDLr knockout mice on a high-fat/high- cholesterol Western-type diet or after dietary lipid lowering. Despite no impact on plasma total cholesterol levels, the infiltration of circulating monocytes into peripheral tissues, and the size of atherosclerotic lesions, abrogation of SSAO activity resulted in the stabilization of established lesions as evidenced by the increased collagen contents under both conditions. Moreover, SSAO inactivation decreased Ly6C^high^ monocytosis and lesion macrophage contents in hypercholesterolemic mice, while no effect was observed in mice after normalization of hypercholesterolemia by dietary lipid lowering. Strikingly, abrogation of SSAO activity significantly increased not only the absolute numbers of smooth muscle cells (SMCs), but also the percent of SMCs with a synthetic phenotype in established lesions of mice regardless of plasma cholesterol levels. Overall, our data indicate that SSAO inactivation *in vivo* stabilizes the established plaques mainly *via* inducing the switch of SMCs from a contractile to a synthetic phenotype. Targeting SSAO activity thus may represent a potential treatment for patients with atherosclerosis.

## Introduction

Atherosclerosis is a chronic inflammatory vascular disease due to the invasion and accumulation of leukocytes in intima. Acute manifestations of atherosclerosis are acute myocardial infarction and stroke that lead to the major morbidity and mortality in the world. The rapid growth and rupture of established lesions are the main mechanisms responsible for acute syndromes [1]. Currently, lowering plasma LDL cholesterol (LDL-C) levels using statins, inhibitors of the *de-novo* cholesterol synthesis, is the main therapeutic strategy to prevent the progression of atheroslcerosis. Although the extent of LDL-C lowering is negatively associated with the rate of atherosclerosis progression [2], a considerable proportion of patients still showed rapid progression of atherosclerosis and the occurrence of clinical events after normalization of hypercholesterolemia by intensive lipid lowering therapy [3, 4]. As such, new therapies are clearly needed for patients with cardiovascular diseases.

Semicarbazide-sensitive amine oxidase (SSAO), also known as vascular adhesion protein-1 (VAP-1) [5], is a difunctional protein. Like the semicarbazide-insensitive monoamine oxidase (MAO), SSAO can deaminate short-chain primary amines (i.e. methylamine and aminoacetone) to generate toxic formaldehyde and methylglyoxal as well as hydrogen peroxide and ammonium. These toxic products could induce protein cross-linkage, oxidative stress and cell death that could all contribute to the development/progression of atherosclerosis [6, 7]. Moreover, both soluble and endothelial VAP-1/SSAO are involved in leukocyte trafficking during inflammation [8, 9], and the enzymatic activity of SSAO/VAP-1 is required for leukocyte extravasation through endothelium [10, 11]. Importantly, serum SSAO activity is positively correlated with the severity of atherosclerosis in clinical studies [12, 13]. Based on these findings, we speculated that modulation of SSAO activity may be a potential therapeutic target for atherosclerosis.

Atherosclerosis may occur in childhood with no symptoms but becomes symptomatic after 40 years old. Manipulation of established lesions is thus of more clinical significance and relevance than the prevention of lesion initiation. To mimic clinical conditions, we herein investigated the effects of SSAO inactivation on the established atherosclerotic plaques under hypercholesterolemia or after normalization of hypercholesterolemia by diet lipid lowering. Our data revealed that SSAO inactivation stabilizes the established atherosclerotic lesions, which is associated with an augmented phenotypic switch of SMCs under both hypercholesterolemic and normocholesterolemic conditions. Therefore, abrogation of SSAO activity may reduce the plaque vulnerability and represent a potential treatment for atherosclerosis.

## Materials and Methods

### Animals

All experiments were performed in accordance with the protocols (*Permit Number*: *201309081*, *201312281*, *20150915*, and *20150925*) approved by the Animal Care and Use Committee of Soochow University. LDL receptor knockout (LDLr KO) mice, obtained from the Jackson Laboratory (Bar Habor, USA), were maintained on sterilized regular chow, containing 4.3% (w/w) fat and no added cholesterol, under specific-pathogen-free (SPF) conditions. At the age of 10 weeks, mice received a Western-type diet (WTD) containing 21% (w/w) total fat and 1.5% cholesterol (Research Diet, USA) for 6 weeks (n = 30, female) or 9 weeks (n = 28, male) to induce the development of atherosclerotic lesions. Thereafter, mice were given the weekly-changed drinking water containing 0.125% semicarbazide hydrochloride (Sigma) to inactivate SSAO *in vivo*. Female mice (15/group) were kept on WTD for another 3 weeks to maintain the hypercholesterolemic condition, while the male ones (14/group) were switched to regular chow diet for the subsequent 6 weeks to lower/normalize the plasma cholesterol levels. Body weight of mice was measured every week and mice with weight loss of 10% or more in one week were euthanized. During the experiments, no animal became ill or died. At the end of each experiment, mice were anaesthetized by subcutaneous injection with a mix of 70 mg/kg body weight xylazine, 1.8 mg/kg bodyweight atropine and 350 mg/kg body weight ketamine. Animals were subsequently sacrificed by cervical dislocation.

### SSAO activity analyses

On sacrifice the arterial tree was perfused *in situ* with PBS before collecting aorta and abdominal fat. Tissue samples were homogenized in ice-cold HES buffer (20 mM HEPES, 1 mM EDTA, sucrose 250 mM, 1x proteases and phosphatases inhibitor (Roche), pH 7.4) prior to assay. The enzymatic activity of SSAO was determined by measuring the production of hydrogen peroxide using fluoropolarimetric assay according to the manufacturer’s instructions (Cell Technology). Background values were obtained from assays performed in the presence of 1 μM of the inhibitor mofegiline.

### Lipid Analyses

After a 4-hour fasting-period, 100 μL of blood was drawn from the mice by tail bleeding. The concentrations of cholesterol in serum were determined using enzymatic colorimetric assays, by incubation with 0.03 U/mL cholesterol oxidase, 0.065 U/mL peroxidase, and 15 μg/mL cholesteryl esterase (all from CalBiochem, Merck, Germany) in reaction buffer (1.0 KPi buffer, pH = 7.7 containing 0.01 M phenol, 1 mM 4-amino-antipyrine, 1% polyoxyethylene-9-laurylether, and 7.5% methanol).

### Flow Cytometry

At sacrifice, blood, spleen, and peritoneal leukocytes were collected/isolated. Single cell suspensions were obtained by squeezing the organs through a 70 μm cell strainer (BD Biosciences), followed by the treatment with erythrocyte lysis buffer. Subsequently, cells were stained for surface markers CD3, CD11b, Ly6G, and Ly6C, or corresponding isotype controls (all from Miltenyi Biotec) for 30 min at 4°C in PBS with 1% mouse serum. Aortic cells were isolated using enzymatic digestion (125 U/ml Collagenase type XI, 60 U/ml Hyaluronidase type 1-s, 450 U/ml Collagenase type I, all from Sigma) after removing the adventitia of aortas, and subsequently stained with antibodies against CD45, α-actin and Ki67, or corresponding isotype controls (all from eBiosciences). After washing, cells were subjected to FACS analysis with FacsCalibur and data were analyzed with CellQuest Pro software (Becton Dickinson, San Jose).

### Histological Analyses of the Aortic Root

On sacrifice the arterial tree was perfused *in situ* with PBS and the heart was excised and stored in 3.7% neutral-buffered formalin (Formal-fixx; Shandon Scientific) until use. Atherosclerotic lesion development was quantified in the aortic root from Oil-red-O/hematoxylin-stained cryostat sections using the Nikon image analysis system with a Nikon CiS microscope coupled to a video camera DS-Fi1 and NIS Elements Imaging software (Nikon Ltd). Mean lesion area (in μm^2^) was calculated from 10 Oil-red-O/hematoxylin-stained sections, starting at the appearance of tricuspid valves. Collagen in lesions was visualized with Sirius Red F3B dye or aniline blue by using Masson’s Trichrome accustain according to the manufacturer’s instructions (Sigma). Sections were immunolabeled with antibodies against MOMA-2 (AbD Serotec), α-actin (Sigma), and Ki67 (Novus Biologicals) for detection of macrophages, SMCs, and proliferative cells, respectively. Necrotic core areas were quantified on Masson’s Trichrome-stained sections. Fibrous cap thickness was quantified by choosing the largest necrotic core from five sections and taking a measurement from the thinnest part of the cap which was determined by measuring the area between the outer edge of the cap and the necrotic core boundary. All analyses were performed double-blinded.

### Vascular SMC (VSMC) Isolation and Culture

Aortas were harvested and the adventitias were removed after enzyme (1 mg/ml Collagenase II and elastase, all from Worthington) treatment. Subsequently, the aortas were cut longitudinally to expose the luminal portion of the vessel and the endothelial cells were removed by gently scraping. The aortas were then placed on gelatin-coated culture dishes and firmly pinned to the base of the culture dish using sterile needles. The explants were cultured in DMEM containing 10% FCS at 37°C and 5% CO_2_. The outgrown VSMCs were collected after 10 days and the purity (>98%) was assessed by flow cytometry using anti-mouse SM α-actin^Alexa 488^ antibody (eBiosciences). VSMCs were subcultured every 7 days before confluence, and experiments were performed between the 4^th^ and 10^th^ passages.

### VSMC Scratch Wound Assay

VSMCs were serum starved (0% FCS) for 16 hours. A linear wound was then gently introduced in the center of the cell monolayer using a 200 μl tip. Cells were then stimulated with mouse PDGF-BB at a final concentration of 20 ng/ml for an additional 24-hour. Images were captured with Olympus FSX100.

### VSMC Proliferation Assay *In Vitro*

VSMCs were serum starved (0% FCS) for 16 hours, and then stimulated with 10 ng/ml mouse PDGF (platelet derived growth factor)-BB (Peprotech) for 48 hours. Edu (10 mg/mL) was added 4 hours before harvesting. Cells were then fixed and stained with Apoll^Alexa 488^ according to the manufacturer’s instructions (RiboBio). The percentages of EdU-positive VSMCS were determined by flow cytometry.

### Collagen Production Assay

VSMCs were extensively washed with PBS and fixed for 30 min with 4% PFA. Cellular collagen production was assessed by staining with Sirius Red F3B dye and the concentrations were determined spectrometrically at 550 nm.

### Statistical Analysis

Statistical analysis was performed using GraphPad InStat and Prism software (Student *t* test or ANOVA and the student-Newman-Keuls post-test). Probability values <0.05 were considered significant.

## Results

### SSAO inactivation stabilized the established atherosclerotic lesions under hypercholesterolemia

**Fig 1:**
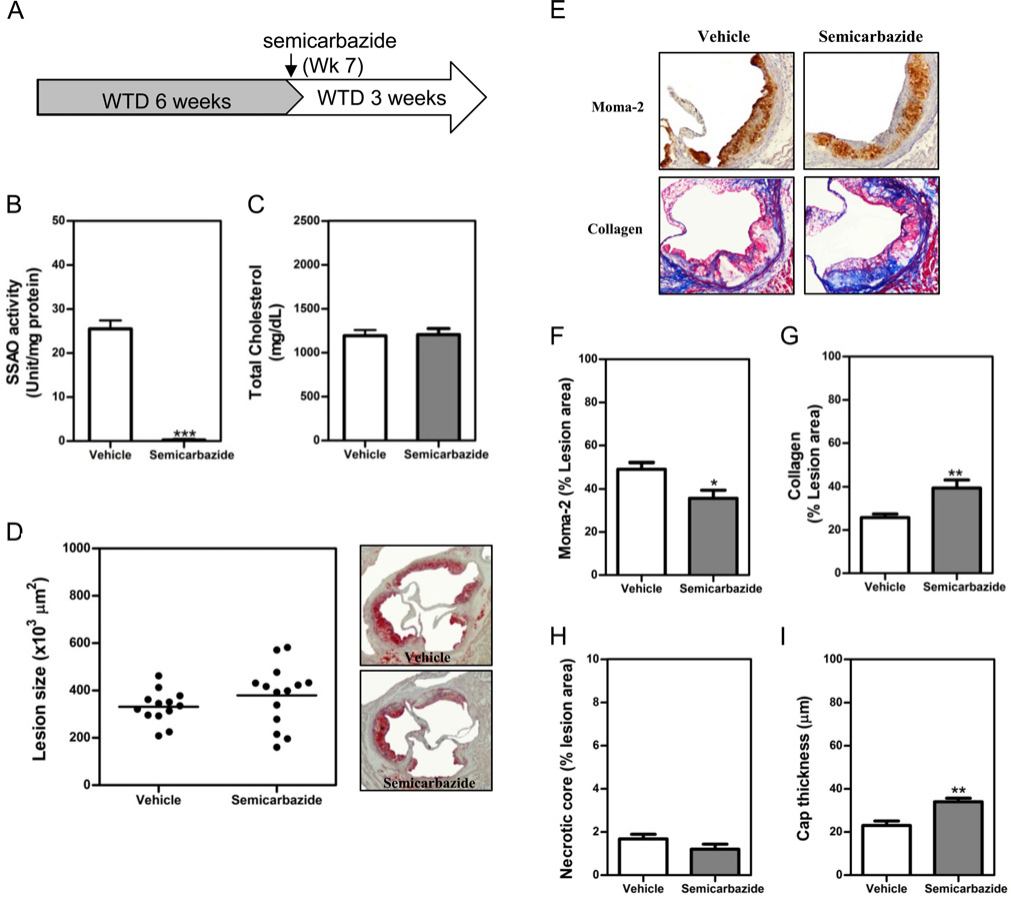
SSAO inactivation stabilized the established atherosclerotic lesions under hypercholesterolemia. Female LDLr KO mice were fed WTD for 6 weeks to induce the formation of atherosclerotic lesions. Thereafter, 0.125% semicarbazide were added in drinking water to inactivate SSAO during the subsequent 3 weeks (A) before the analysis of SSAO activities in the visceral adipose tissue (B), plasma cholesterol levels (C) and atherosclerotic lesions at the aortic root (D-G). (D) A scattered dot plot (*left*) or representative photomicrographs showing the size of atherosclerotic lesions. Sections were stained with oil-red-O (original magnification 40x, *right panel*). Each dot represents the mean lesion area of a single mouse, and the horizontal bar indicates the mean value of the group (*left panel*). (E) Representative photomicrographs showing macrophages and collagens in lesions. Sections of aortic roots were stained with antibodies against Moma-2 to visualize macrophages (brown, 100x) or with Masson’s Trichrome Accustain to visualize collagens (blue, 100x). (F & G) Bar graphs showing the lesion contents of macrophages (F), collagens (G), necrotic core (H) and cap thickness (I). Results were expressed as mean±SEM. Statistically significant difference *p<0.05, **p<0.01, and ***p<0.001 *vs* vehicle.

Female LDLr-/- mice were fed WTD for 6 weeks to induce the formation of atherosclerotic lesion. Thereafter, semicarbazide was added in drinking water to inactivate SSAO during the subsequent 3 weeks (Fig 1A). Based on the volumes of water consumed, the mean daily intake of semicarbazide is 62.5–75 mg/kg, significantly lower than the oral LD50 (225 mg/kg) reported in mice [14]. Semicarbazide drinking completely eliminated the SSAO activity of visceral adipose tissues (Fig 1B) and aortas (S1A Fig). However, it did not affect the high levels of total plasma cholesterol in animals fed WTD diet (Fig 1C). Despite the comparable lesion sizes in vehicle- and semicarbazide-treated mice (Fig 1D), a significantly reduced macrophage content (1.4-fold, p<0.05) (Fig 1E and 1F) while increased accumulation of collagens (1.5-fold, p<0.05) (Fig 1E and 1G and S2 Fig) were observed in lesions of mice with inactivated SSAO (Fig 1E–1G). SSAO inactivation also led to a 1.5-fold (p<0.01) increase in the cap thickness (Fig 1I) of lesions, despite no effect on the content of necrotic core (Fig 1H). These data indicated that abrogation of SSAO activity stabilizes the established atherosclerotic lesions, although it hardly affects total plasma cholesterol levels and the lesion size.

### SSAO inactivation did not inhibit the recruitment of monocytes from the circulation into peripheral tissues under hypercholesterolemia

**Fig 2:**
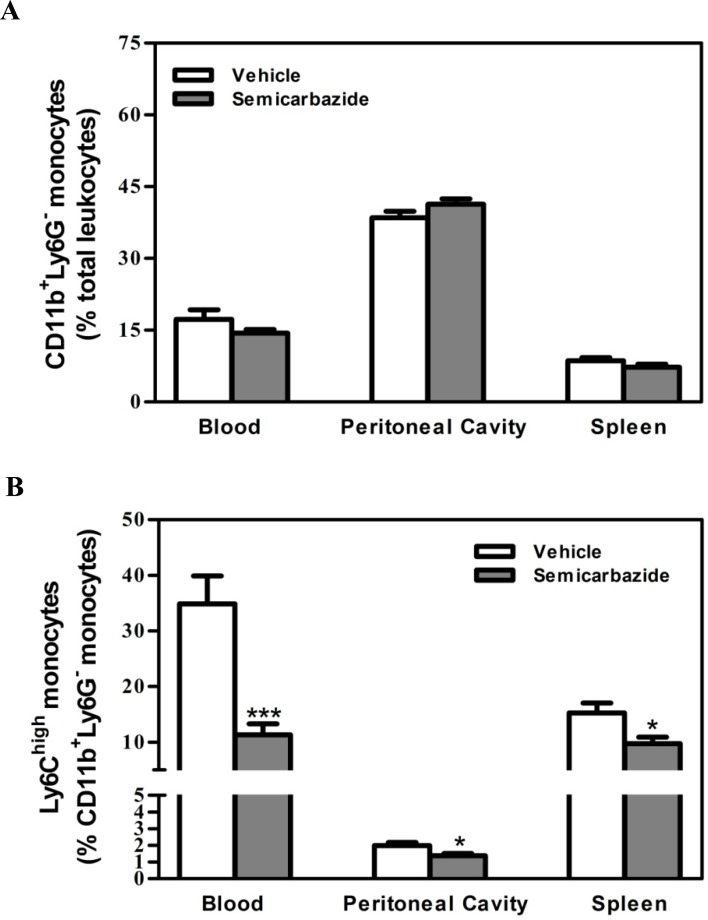
Effect of SSAO inactivation on the migration of monocytes under hypercholesterolemia. Female LDLr KO mice were treated as described in the legend to Fig 1. Upon sacrifice, the percentage of CD11b^+^Ly6G^-^ (A) and CD11b^+^Ly6G^-^Ly6C^high^ monocytes (B) in the blood, peritoneal cavity, and spleen were analyzed by flow cytometry. Comparable absolute numbers of total cells were obtained in corresponding tissues of mice from each group. Results were expressed as mean±SEM. Statistically significant difference *p<0.05 and ***p<0.001 *vs* vehicle.

Given that the enzymatic activity of SSAO/VAP-1 is required for leukocyte extravasation through endothelium [10, 11], the reduction of macrophage content in lesions of semicarbazide-treated mice could be attributed to the decreased infiltration of circulating monocytes. We thus compared the leukocyte cell counts in the circulation, peritoneal cavity and spleen of mice treated with or without semicarbazide to determine the effect of SSAO inactivation on their migration. No differences were observed on the absolute number of cells collected from corresponding tissues in both groups of mice (data not shown). As expected, a reduced migration of CD3^+^ T lymphocytes (0.80-fold, p<0.05) and CD11b^+^Ly6G^+^ neutrophils (0.78-fold in the peritoneal cavity and 0.67-fold in the spleen, p<0.05) from circulations into the peripheral tissues were observed in mice with abrogated SSAO activity (S3 Fig). By contrast, comparable amounts of monocytes were obtained among corresponding tissues in control and semicarbazide-treated mice (Fig 2A). These data indicate that the migration of T cells and neutrophils, but not monocytes, from the circulation to peripheral tissues was inhibited by SSAO inactivation under hypercholesterolemia. Thus, it is unlikely that the reduced lesion macrophages in semicarbazide-treated mice were attributed to the decreased influx of circulating monocytes. Notably, a significant lower amount of Ly6C^high^ monocytes in the blood (0.32-fold, p<0.001), peritoneal cavity (0.70-fold, p<0.05) and spleen (0.64-fold, p<0.05) was observed in mice treated with semicarbazide (Fig 2B).

### SSAO inactivation increased the percent of SMCs with a synthetic phenotype in lesions of mice with hypercholesterolemia

**Fig 3:**
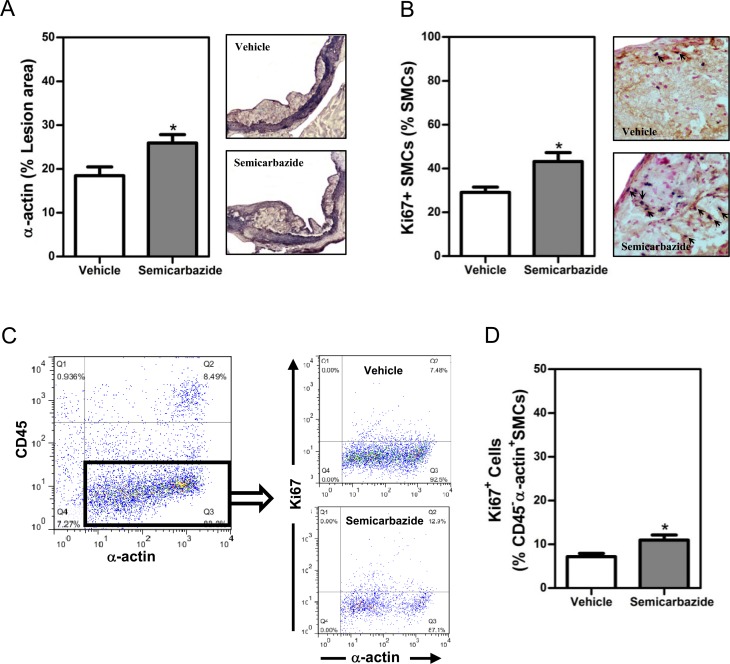
SSAO inactivation increased the percent of synthetic SMCs *in* established lesions under hypercholesterolemia. Female LDLr KO mice were treated as described in the legend to Fig 1. (A) A bar graph (*left*) and representative photomicrographs (*right*) showing the lesion contents of SMCs. (B) A bar graph (*left*) or representative photomicrographs (*right*) showing the lesion contents of SMCs positive for Ki67. Sections of the aortic root were stained with the antibody against α-actin in the absence (A, purple for α-actin, 100x) or presence of anti-Ki67 (B, brown for α-actin while nuclear dark blue for Ki67, indicated by arrows, 400x) to visualize total or proliferative SMCs, respectively. Nuclei were counterstained by fast nuclear red (nuclear pink, B). (C) Dot plots showing proliferative VSMCs. Aortic cells were stained with antibodies against CD45, α-actin, and Ki67. The percent of proliferative (Ki67^+^) SMCs among α-actin^+^CD45^-^ aortic cells are analyzed. (D) Bar graph showing the percent of proliferative (Ki67^+^) SMCs. Results were expressed as mean±SEM. Statistically significant difference *p<0.05 *vs* vehicle.

In addition to reduced MMP-secreting macrophages, increased amount of collagen-producing SMCs in lesions could result in the augmented accumulation of collagens as well. By α-actin staining, we indeed observed greater amounts of SMCs (1.4-fold, p<0.05) in lesions of mice with inactivated SSAO (Fig 3A). Considering the toxicity of SSAO-mediated deamination, we reasoned that increased lesion SMCs may result from the reduced apoptosis of SMCs by elimination of SSAO activity. However, comparable numbers of lesion apoptotic SMCs were observed in lesions of vehicle- or semicarbazide-treated mice by TUNEL staining (S4 Fig).

Before migration, SMCs need to switch from a contractile to a synthetic phenotype, a process called phenotype switch. Moreover, SMCs with a synthetic phenotype harbor increased proliferative abilities as well. The increased phenotypic switch of SMCs could thus lead to enhanced accumulation of lesion SMCs in SSAO inactivated mice. Therefore, we next determined the percent of Ki67^+^ SMCs to visualize the proliferating SMCs. As shown in Fig 3B, the percentage of Ki67^+^ SMCs was significantly increased (1.5-fold, p<0.05) in lesions of mice with inactivated SSAO (Fig 3B). The increased percent (1.5-fold, p<0.05) of proliferative Ki67^+^ SMC was observed in aortas of semicarbazide-treated mice by flow cytometric analysis as well (Fig 3C).

Together, these data indicate that inactivation of SSAO by semicarbazide induces SMCs to switch from a contractile to a synthetic phenotype with increased migratory and proliferative ability, thereby contributing to the accumulation of SMCs and collagens in established lesions under hypercholesterolemia.

### SSAO inactivation stabilized the established atherosclerotic lesions after normalization of hypercholesterolemia by diet lipid lowering

**Fig 4:**
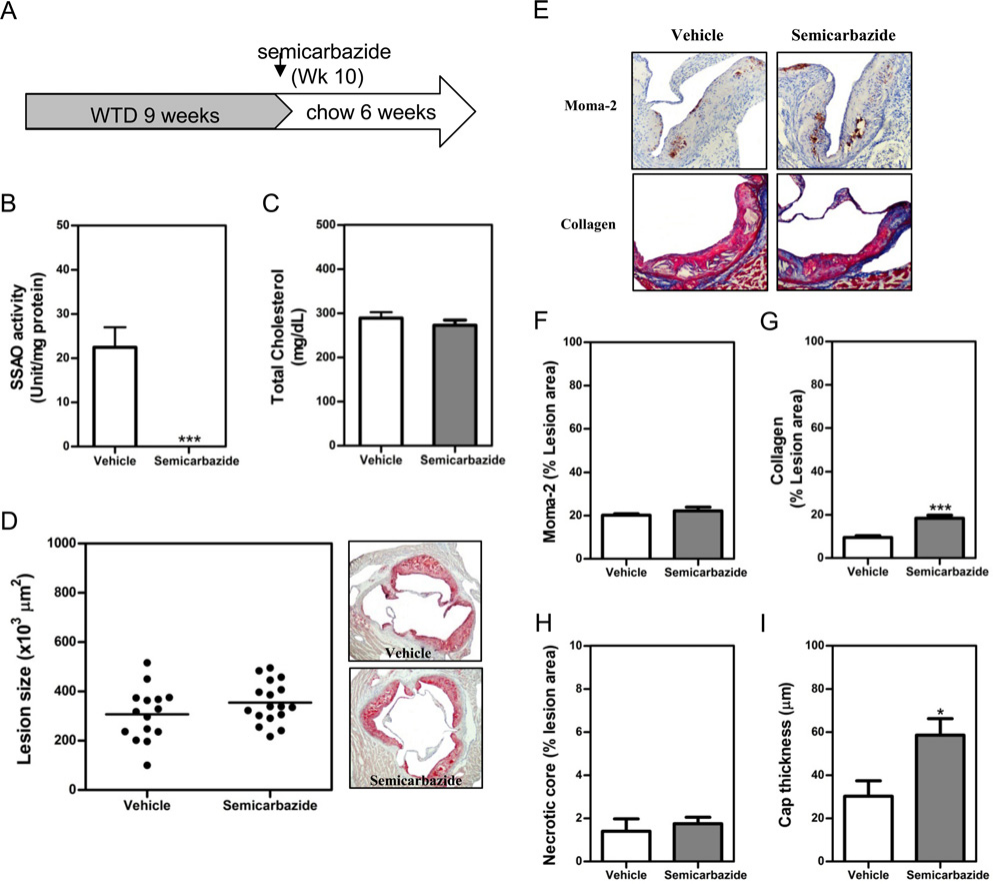
SSAO inactivation stabilized the established atherosclerotic lesions after normalization of hypercholesterolemia by diet lipid lowering. Male LDLr KO mice were fed WTD for 9 weeks to induce the formation of atherosclerotic lesions. Thereafter, these animals were fed regular chow diet to normalize hypercholesterolemia (A). Drinking water containing 0.125% semicarbazide was given to these chow-fed animals in the subsequent 6 weeks (A) before the analysis of SSAO activities in the visceral adipose tissue (B), plasma cholesterol levels (C) or atherosclerotic lesions at the aortic root (D-G). (D) A scattered dot plot (*left*) or representative photomicrographs showing the size of atherosclerotic lesions. Sections were stained with oil-red-O (original magnification 40x, *right panel*). Each dot represents the mean lesion area of a single mouse, and the horizontal bar indicates the mean value of the group (*left panel*). (E) Representative photomicrographs showing the lesion contents of Moma-2^+^ macrophages (*upper panel*) or collagens (*lower panel*) in each group. Sections of aortic roots were stained with antibodies against Moma-2 to visualize macrophages (brown, 100x) or with Masson’s Trichrome Accustain to visualize collagens (blue, 100x). (F & G) Bar graphs showing the lesion contents of macrophages (F), collagens (G), necrotic core (H), and cap thickness (I) in vehicle- or semicarbazide-treated mice. Results were expressed as mean ±SEM. Statistically significant difference ***p<0.001 and *p<0.05 *vs* vehicle.

Currently, lipid lowering to normalize hypercholesteroemia by statins is a routine clinical treatment to prevent the rapid progression and induce stabilization of established atherosclerotic plaques in patients with coronary diseases. Therefore, we wondered whether SSAO inactivation is still beneficial for established plaques when combined with lipid lowering. To this end, after the induction of atherosclerotic lesions in male LDLr-/- mice by WTD for 9 weeks, animals were switched to chow diet to normalize the plasma cholesterol levels (Fig 4A). Simultaneously, a group of mice were treated with 0.125% semicabazide for 6 weeks to eliminate the activity of SSAO. Based on the volumes of water consumed, the daily intake of semicabazide by male mice is only 47.5–56.8 mg/kg, significantly lower than that consumed by female mice. Nonetheless, the SSAO activity in adipose tissues (Fig 4B) and aorta (S1B Fig) was completely abrogated in semicabazide-treated mice. In line with the results obtained in hypercholesterolemic mice, no significant difference in total plasma cholesterol levels (both are 4.1-fold lower compared to those in WTD feeding, p<0.001) or atherosclerotic lesion size was observed between vehicle- and semicarbazide-treated mice after dietary lipid lowering (Fig 4C and 4D). Nevertheless, a significantly enhanced accumulation of lesion collagens (1.9-fold, p<0.05) (Fig 4E & 4G) and an increased thickness of lesion cap (1.9-fold, p<0.05) (Fig 4I) were observed in mice with inactivated SSAO, reflecting an increased stability of established lesions. Notably, the amounts of lesion macrophages (Fig 4E and 4F) and necrotic core (Fig 4H) were comparable between vehicle- and semicarbazide-treated mice. Likewise, on a regular chow diet, SSAO inactivation did not impact the counts of either total (S5A Fig) or Ly6C^high^ monocytes (S5B Fig) in the circulation and peripheral tissues.

### SSAO inactivation increased the percent of SMCs with a synthetic phenotype in lesions of mice with normacholesterolemia after diet lipid lowering

**Fig 5:**
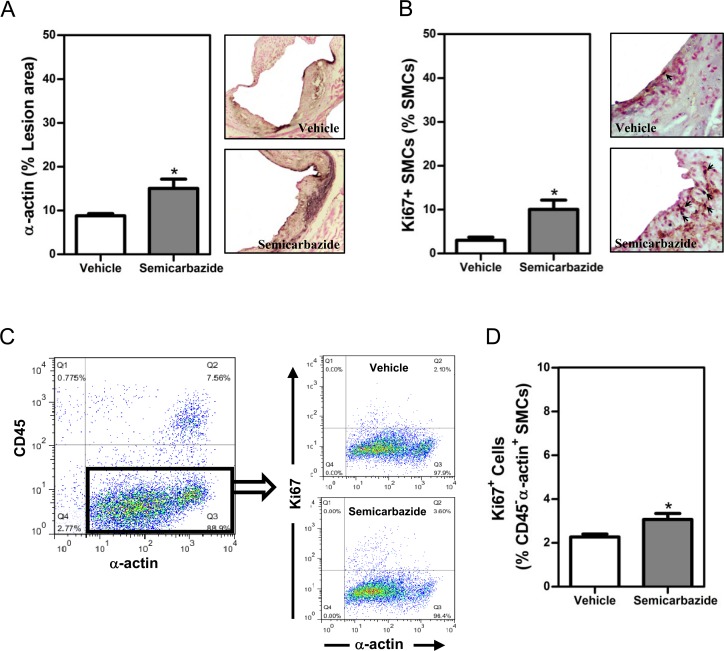
SSAO inactivation increased the synthetic SMCs in established lesions under normacholesterolemia after dietary lipid lowering. Male LDLr KO mice were treated as described in the legend to Fig 4. (A) A bar graph (*left*) or representative photomicrographs (*right*) showing the lesion contents of SMCs. (B) A bar graph (*left*) or representative photomicrographs (*right*) showing the lesion contents of SMCs positive for Ki67 in vehicle- or semicarbazide-treated mice. Sections of the aortic root were stained with the antibody against α-actin in the absence (A, purple for α-actin, 100x) or presence of anti-Ki67 (B, red for α-actin while nuclear dark blue for Ki67, indicated by arrows, 400x) to visualize total or proliferative SMCs, respectively. Nuclei were counterstained by fast nuclear red (nuclear pink, B). (C) Dot plots showing proliferative VSMCs. Aortic cells were stained with antibodies against CD45, α-actin, and Ki67. The percent of proliferative (Ki67^+^) SMCs among α-actin^+^CD45^-^ aortic cells are analyzed. (D) Bar graph showing the percent of proliferative (Ki67^+^) SMCs. Results were expressed as mean±SEM. Statistically significant difference *p<0.05 *vs* vehicle.

To further confirm the effect of SSAO inactivation on the number and phenotype of lesion SMCs when combined with lipid lowering treatment, we analyzed the amount of total SMCs as well as the percent of cells positive for Ki67 in lesions of these normocholesterolemic mice after dietary lipid lowering. Indeed, a significantly increased (1.7-fold, p<0.05) amount of SMCs in atherosclerotic lesions of semicarbazide-treated mice was observed (Fig 5A), indicating that SSAO inactivation enhances the migratory capacity of SMCs under normocholesterolemia as well. Moreover, SSAO inactivation increased the proliferative capacity of SMCs as evidenced by the increased percent of Ki67^+^ SMCs in both lesions (3.3-fold, p<0.05, Fig 5B) and aortas (1.4-fold, p<0.05, Fig 5C) of semicarbazide-treated mice. Collectively, these data indicate that elimination of SSAO activity promotes a synthetic phenotype of SMCs in established lesions under normocholesterolemia after dietary lipid lowering.

### Soluble SSAO inhibits the phenotype switch of VSMCs *in vitro*

**Fig 6:**
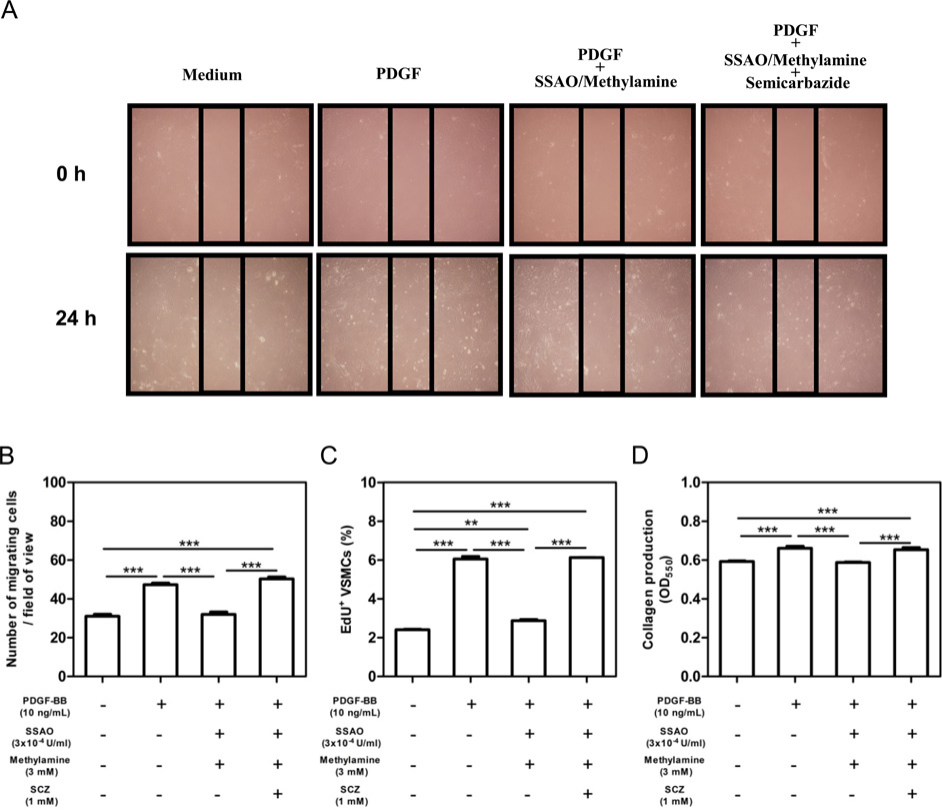
Soluble SSAO inhibited the phenotype switch of VSMCs *in vitro*. Murine VSMCs were starved and stimulated with 20 ng/mL murine PDGF-BB to induce a phenotype switch in the absence/presence of soluble bovine SSAO and methylamine together with/without semicarbazide for 24 hours. (A) Representative pictures of VSMC migration through scratch-wound. (B) Bar graph showing the quantification of migrated cell numbers under field of view. The average number of migrated cells was determined by counting 6 fields of view per well. (C) Bar graph showing the percent of EdU incorporated proliferative VSMCs determined by flow cytometric analysis. (D) Bar graph showing collagen productions of VSMCs. Values represent the mean ±SEM (n = 4 wells). Statistically significant difference ***p<0.001 and **p<0.01 *vs* medium.

To further confirm the *in-vivo* effect of SSAO inactivation on the phenotypic switch of SMCs, VSMCs from wild-type mice were generated. SSAO enzymatic activity was not detectable in murine VSMCs, despite the presence of protein expression (data not shown). Therefore, a soluble bovine SSAO was used to determine the effect of SSAO enzymatic activity on PDGF-induced mobilizations, proliferations, and collagen productions of VSMCs. As shown in Fig 6, murine PDGF-BB (20 ng/mL) increased the mobilization (1.5-fold, p<0.001), proliferation (2.5-fold, p<0.001), and collagen production (1.1-fold, p<0.001) of VSMCs. Additions of soluble bovine SSAO (3x10^-4^ U/mL) together with its substrate methylamine (3mM) significantly inhibited the PDGF-induced mobilization (0.7-fold, p<0.001, Fig 6A and 6B), proliferation (0.5-fold, p<0.001, Fig 6C), and collagen production (0.9-fold, p<0.001, Fig 6D). Importantly, these suppressive effects of SSAO plus methylamine was almost completely reversed by semicarbazide (Fig 6). Soluble SSAO and methylamine at the concentration used here did not affect the viability of VSMCs (data not shown). These data thus demonstrated that semicarbazide is capable of releasing the inhibitory effect of SSAO enzymatic activity on the switch of SMCs to a synthetic phenotype *in vitro*.

## Discussion

Ruptures of the plaque surface and the subsequent luminal thrombus formation are thought to be the most important mechanisms underlying the acute syndromes of cardiovascular diseases [15]. Moreover, repeated ruptures also result in a rapid progression of atherosclerotic lesions [16]. As such, stabilization of established atheroma has emerged as an important strategy for the treatment of atherosclerosis. The present study, for the first time to our knowledge, evaluated the therapeutic potential of SSAO inactivation for established atherosclerotic lesions. Our data revealed that SSAO inactivation by semicarbazide significantly increases the accumulation of collagens and the thickness of cap in plaques and thereby stabilizes the established atherosclerotic plaques in mice on WTD or after dietary lipid lowering. Importantly, our data indicated that this effect is mainly mediated by a previously unappreciated novel function of SSAO, regulating the phenotypic switch of SMCs.

Plaque stability appears to be a balance between the mechanical stress on the fibrous cap and the biological factors regulating fibrous cap’s strength [17]. Therefore, plaque composition rather than its size determines the risk of plaque rupture. Accordingly, numerous studies have demonstrated that the modification of plaque cellular compositions, especially the amount of macrophages/SMCs that degradate/synthesize collagens, respectively, determines the stability of atheromas. In line with this notion, we here showed that the increased collagen accumulation and thus enhanced plaque stability is indeed associated with greater amounts of lesion SMCs. Notably, although lipid lowering stimulates the accumulation of lesion SMCs and collagens [18], inhibition of SSAO activity *in vivo* induced a further increase of both in established plaques of mice after diet lipid lowering. Therefore, abrogation of SSAO activity stabilizes atherosclerotic plaques, at least in our animal model, by increasing the contents/numbers of collagens/SMCs under both hypercholesteromic and normocholesteromic conditions.

SSAO-mediated deamination produces a serial of toxic molecules, including formaldehyde and hydrogen peroxide. It is thus conceivable that increased contents of lesion collagens and SMCs in mice with deficient SSAO activity are attributed to the reduced apoptosis of SMCs, the main producers of cap-stabilizing collagen [19, 20]. However, we did not observe a reduced apoptosis of SMCs in lesions by TUNEL staining after the blockade of SSAO. In line with previous findings showing an elevated SSAO/VAP-1 activity during the differentiation of rat aortic SMCs into a contractile phenotype *in vitro* [21], our data here showed that mice with abrogated SSAO activity *in vivo* harbored more synthetic lesion SMCs with enhanced migratory and proliferative capacities both in *vivo* and *in vitro*. Together, these data disclosed a novel function of SSAO, regulating the switch of SMCs between contractile and synthetic phenotypes. Intriguingly, enhanced H_2_O_2_ production in SMCs has been shown to correlate with an acceleration of switch from the contractile to the synthetic phenotype [22]. Nevertheless, the detailed molecular mechanisms underlying the regulation of SMCs phenotypes by SSAO warrant further investigations.

Given that the adhesive function of SSAO requires its enzymatic activity, abrogation of SSAO activity by semicarbazide may inhibit the infiltration of monocytes and subsequently reduce the secretion of metalloproteinases (MMPs) to relieve the weakening burden of plaques. In contrast to the comparable amounts of monocytes in peripheral tissues of mice, irrespective of SSAO activities and cholesterol levels, a decrease in lesion macrophage content was only observed in hypercholesterolemic mice after the abrogation of SSAO. It is thus unlikely that the reduction of lesion macrophage content in SSAO-inactivated hypercholesterolemic mice was attributable to the impaired infiltration of monocytes from circulation. Notably, SSAO inactivation reduced the hypercholesterolemia-induced increase of Ly6C^high^ monocytes. Because Ly6C^high^ monocytes preferentially accumulate in atherosclerotic lesions as compared to their Ly6C^low^ counterparts [23], it is conceivable that the reduced Ly6C^high^ monocytosis by SSAO inactivation results in the decreased lesion macrophage content in hypercholesterolemic mice.

Recently, both human and animal studies have demonstrated that VSMCs are the main origins of cells contributing to the development of atherosclerotic plaques [24, 25]. After migration into atherosclerotic lesions, vascular SMCs could further transdifferentiate into macrophages, which subsequently take up accumulated lipids to become foam cells, the hall marker of atherosclerosis [26]. However, despite the increased SMCs in lesions of semicarbazide-treated mice, the amount of lesion macrophages were not accordingly increased, indicating that SSAO inactivation inhibits the transdifferentiation of SMCs into macrophages. More importantly, the increased accumulation of lesion SMCs with a switched phenotype in SSAO-inactivated mice resulted in the stabilization, but not the growth of atherosclerotic lesions. As such, targeting SSAO enzymatic activities may represent a therapeutic strategy to stabilize and thereby prevent the rupture of plagues in patients with atherosclerosis.

In conclusion, abrogation of SSAO activity *in vivo* stabilized the established atherosclerotic plaques under hypercholesterolemia or after dietary lipid lowering. Although SSAO inactivation may reduce oxidative stress-induced vascular cell death and hamper the infiltration of leukocytes, our data indicate that the increased plaque stability by SSAO inactivation is mainly attributed to the previously unappreciated inhibitory effect of SSAO on the phenotypic switch of SMCs. SSAO thus represents a potential therapeutic target in atherosclerosis patients.

## Supporting Information

S1 Fig Effect of semicarbazide on the activities of SSAO in the aortas. (A) Female LDLr KO mice were fed WTD for 6 weeks to induce the formation of atherosclerotic lesions. Thereafter, 0.125% semicarbazide were added in drinking water to inactivate SSAO during the subsequent 3 weeks before the analysis of SSAO activities in the aortas. (B) Male LDLr KO mice were fed WTD for 9 weeks to induce the formation of atherosclerotic lesions. Thereafter, these animals were fed regular chow diet to normalize hypercholesterolemia (A). Drinking water containing 0.125% semicarbazide was given to these chow-fed animals in the subsequent 6 weeks before the analysis of SSAO activities in the aortas. Values represent the mean ±SEM. Statistically significant difference ***p<0.001 *vs* vehicle.
(TIF)

S2 Fig SSAO inactivation increases the collagen content in the established atherosclerotic lesions on WTD. (A) Photomicrographs showing Picro Sirius red- and Masson’s trichrome-stained sections. Collagens were stained as red by Masson’s trichrome (1,3, and 5) staining and as blue by Picro Sirius red (2,4, and 6) staining. (B) Photomicrographs showing a scatter dot plot of collagen content in lesions (left panel) and representative Sirius Red-stained sections (right panel, 100x). Female LDLr KO mice were treated as described in the legend to S1A Fig. Sections of aortic roots were stained with Sirius Red F3B dye to visualize collagens. Values represent the mean ±SEM. Statistically significant difference **p<0.01 *vs* vehicle.
(TIF)

S3 Fig SSAO inactivation inhibits the infiltration of T lymphocytes and neutrophils into peripheral tissues. Female LDLr KO mice were treated as described in the legend to S1A Fig. CD3^+^ T lymphocytes (A) and CD11b^+^Ly6G^+^ neutrophils (B) in the blood, peritoneal cavity, and spleen were analyzed by flow cytometry. Values represent the mean ±SEM. Statistically significant difference *p<0.05 *vs* vehicle.
(TIF)

S4 Fig SSAO inactivation did not reduce the cell apoptosis. Female LDLr KO mice were treated as described in the legend to S1A Fig. TUNEL staining was perform to detect the apoptotic cells. Values represent the mean ±SEM.
(TIF)

S5 Fig Effect of SSAO inactivation on the migration of monocytes after diet lipid lowering. Male LDLr KO mice were treated as described in the legend to S1B Fig. Upon sacrifice, the percentage of CD11b^+^Ly6G^-^ (A) and CD11b^+^Ly6G^-^Ly6C^high^ monocytes (B) in the blood, peritoneal cavity, and spleen were analyzed by flow cytometry. Comparable absolute numbers of total cells were obtained in corresponding tissues of mice from each group. Results were expressed as mean±SEM.
(TIF)
