# Supplementary figures and images for: Inactivation of Semicarbazide-Sensitive Amine Oxidase Stabilizes the Established Atherosclerotic Lesions via Inducing the Phenotypic Switch of Smooth Muscle Cells
Source: PLoS One. 2016 Apr 4;11(4):e0152758. doi: 10.1371/journal.pone.0152758 (PMC4820117; doi:10.1371/journal.pone.0152758)

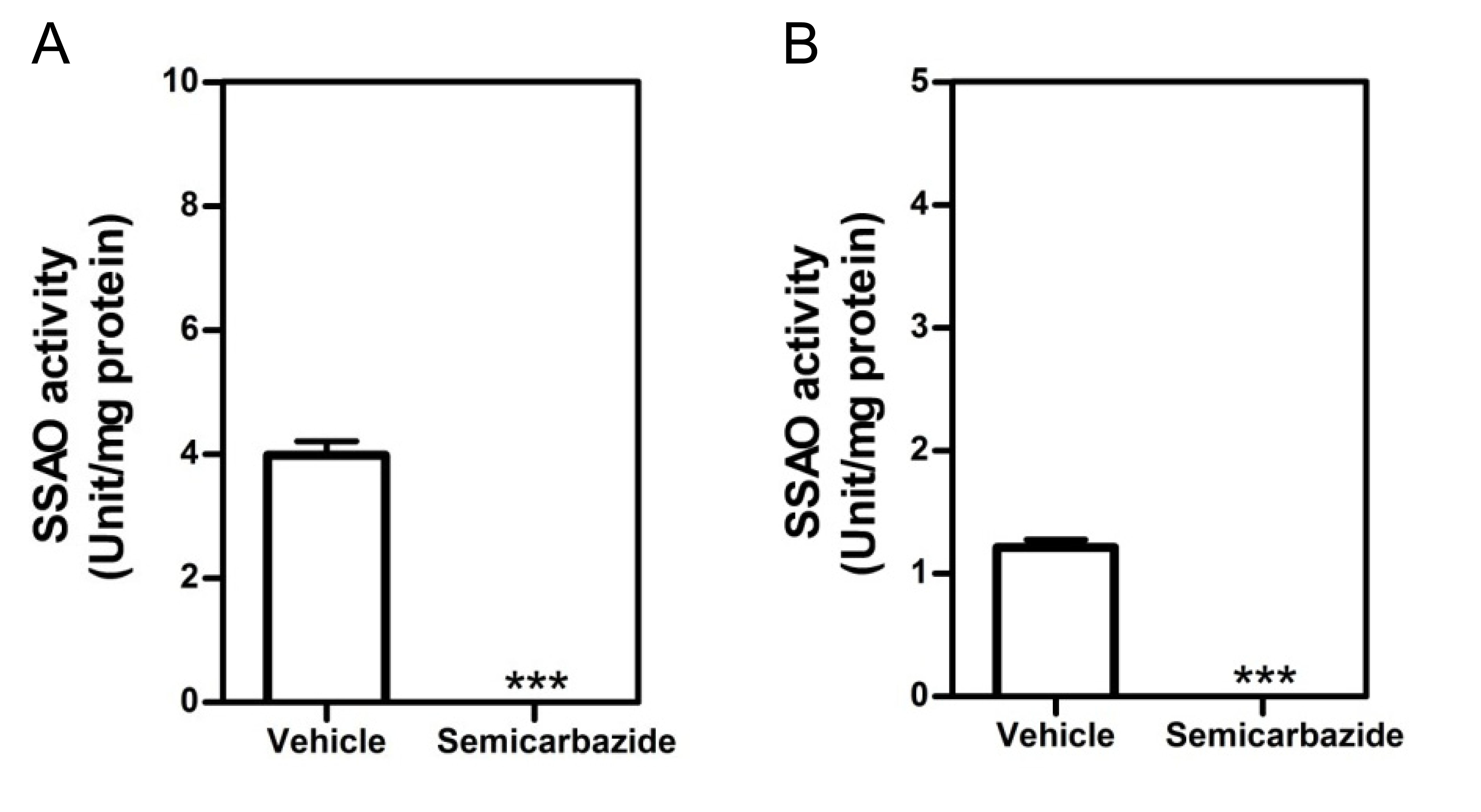

Supplement: S1 Fig — (A) Female LDLr KO mice were fed WTD for 6 weeks to induce the formation of atherosclerotic lesions. Thereafter, 0.125% semicarbazide were added in drinking water to inactivate SSAO during the subsequent 3 weeks before the analysis of SSAO activities in the aortas. (B) Male LDLr KO mice were fed WTD for 9 weeks to induce the formation of atherosclerotic lesions. Thereafter, these animals were fed regular chow diet to normalize hypercholesterolemia (A). Drinking water containing 0.125% semicarbazide was given to these chow-fed animals in the subsequent 6 weeks before the analysis of SSAO activities in the aortas. Values represent the mean ±SEM. Statistically significant difference ***p<0.001 vs vehicle. (TIF) [file pone.0152758.s001.tif]

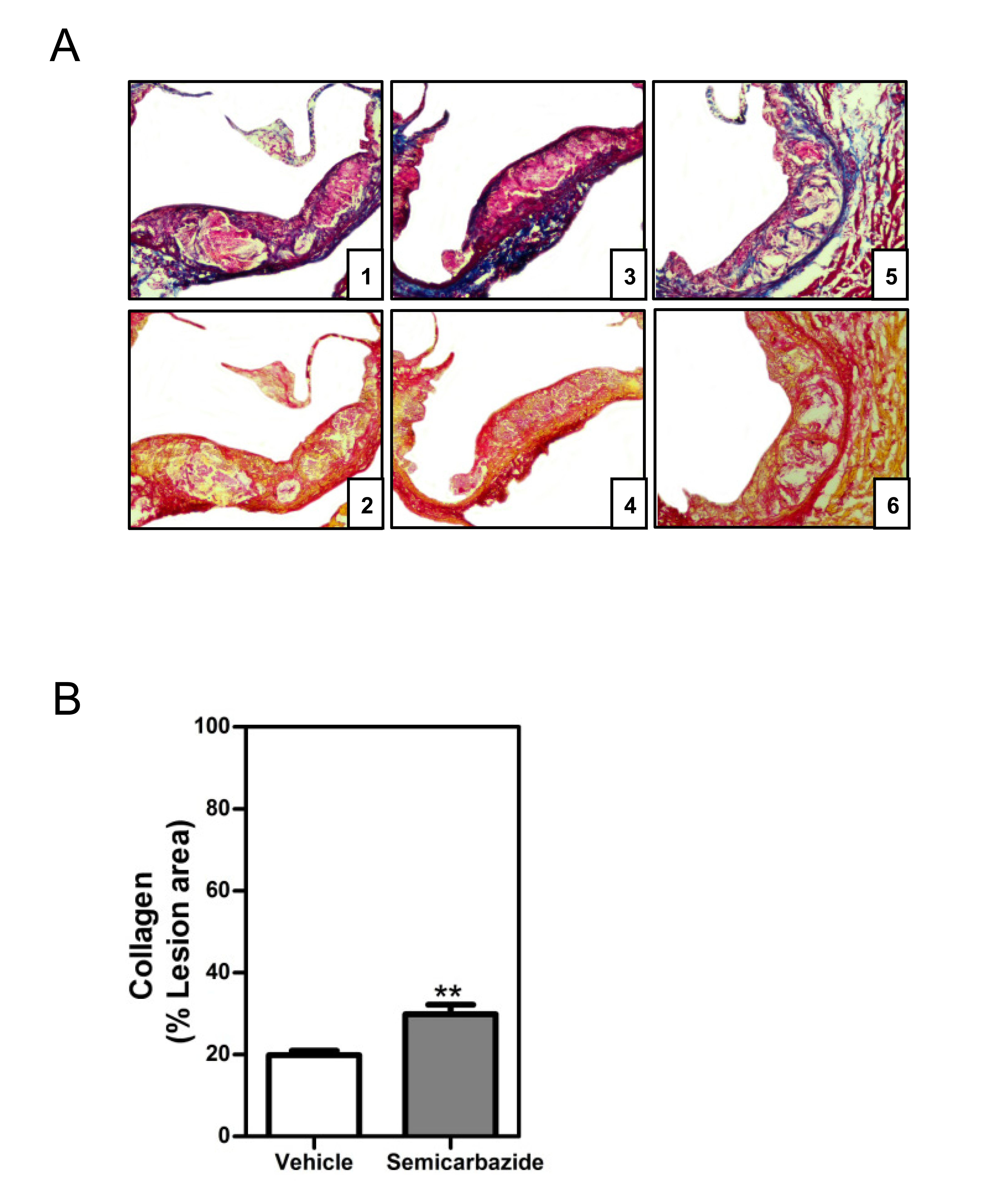

Supplement: S2 Fig — (A) Photomicrographs showing Picro Sirius red- and Masson’s trichrome-stained sections. Collagens were stained as red by Masson’s trichrome (1,3, and 5) staining and as blue by Picro Sirius red (2,4, and 6) staining. (B) Photomicrographs showing a scatter dot plot of collagen content in lesions (left panel) and representative Sirius Red-stained sections (right panel, 100x). Female LDLr KO mice were treated as described in the legend to S1A Fig. Sections of aortic roots were stained with Sirius Red F3B dye to visualize collagens. Values represent the mean ±SEM. Statistically significant difference **p<0.01 vs vehicle. (TIF) [file pone.0152758.s002.tif]

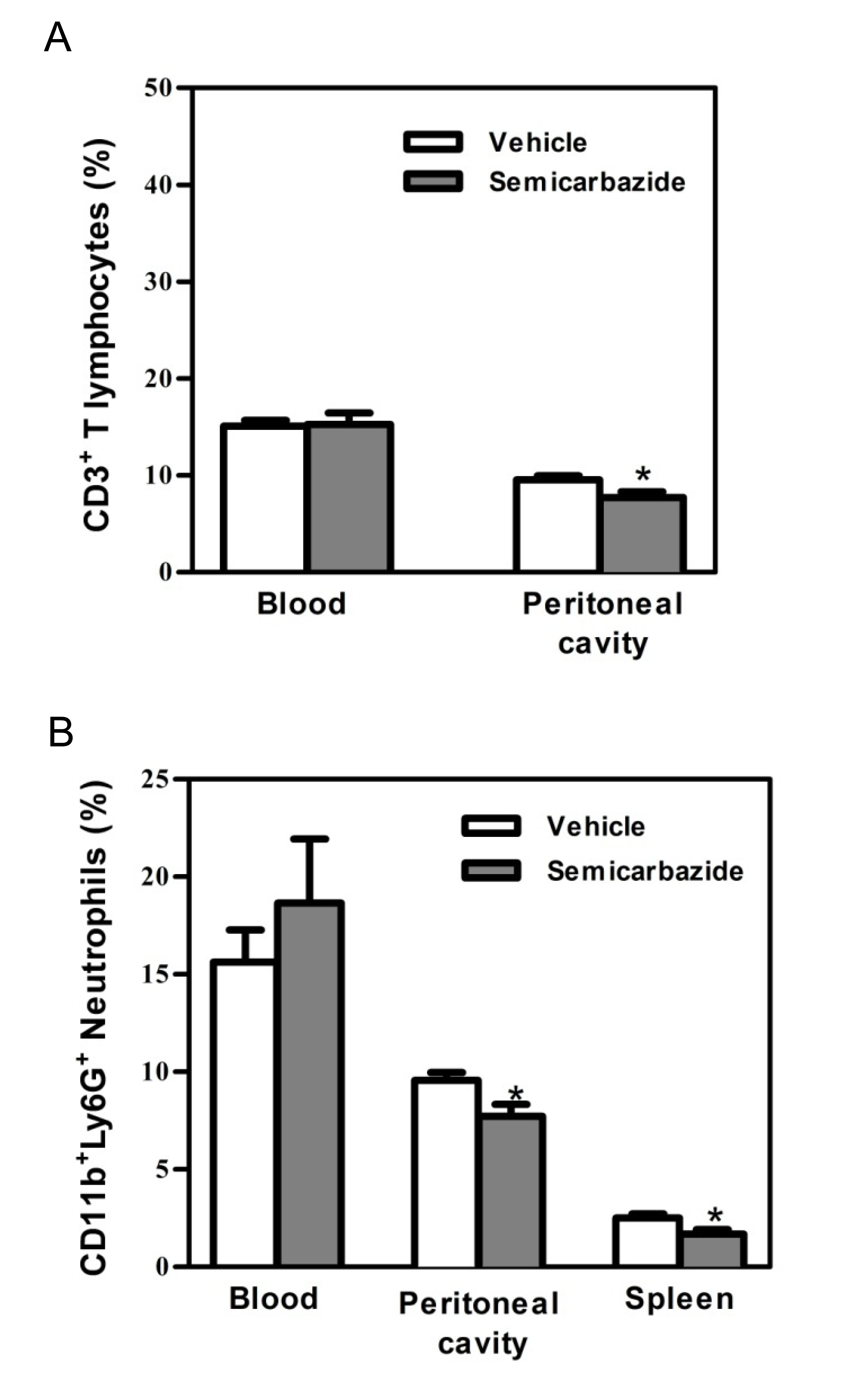

Supplement: S3 Fig — Female LDLr KO mice were treated as described in the legend to S1A Fig. CD3+ T lymphocytes (A) and CD11b+Ly6G+ neutrophils (B) in the blood, peritoneal cavity, and spleen were analyzed by flow cytometry. Values represent the mean ±SEM. Statistically significant difference *p<0.05 vs vehicle. (TIF) [file pone.0152758.s003.tif]

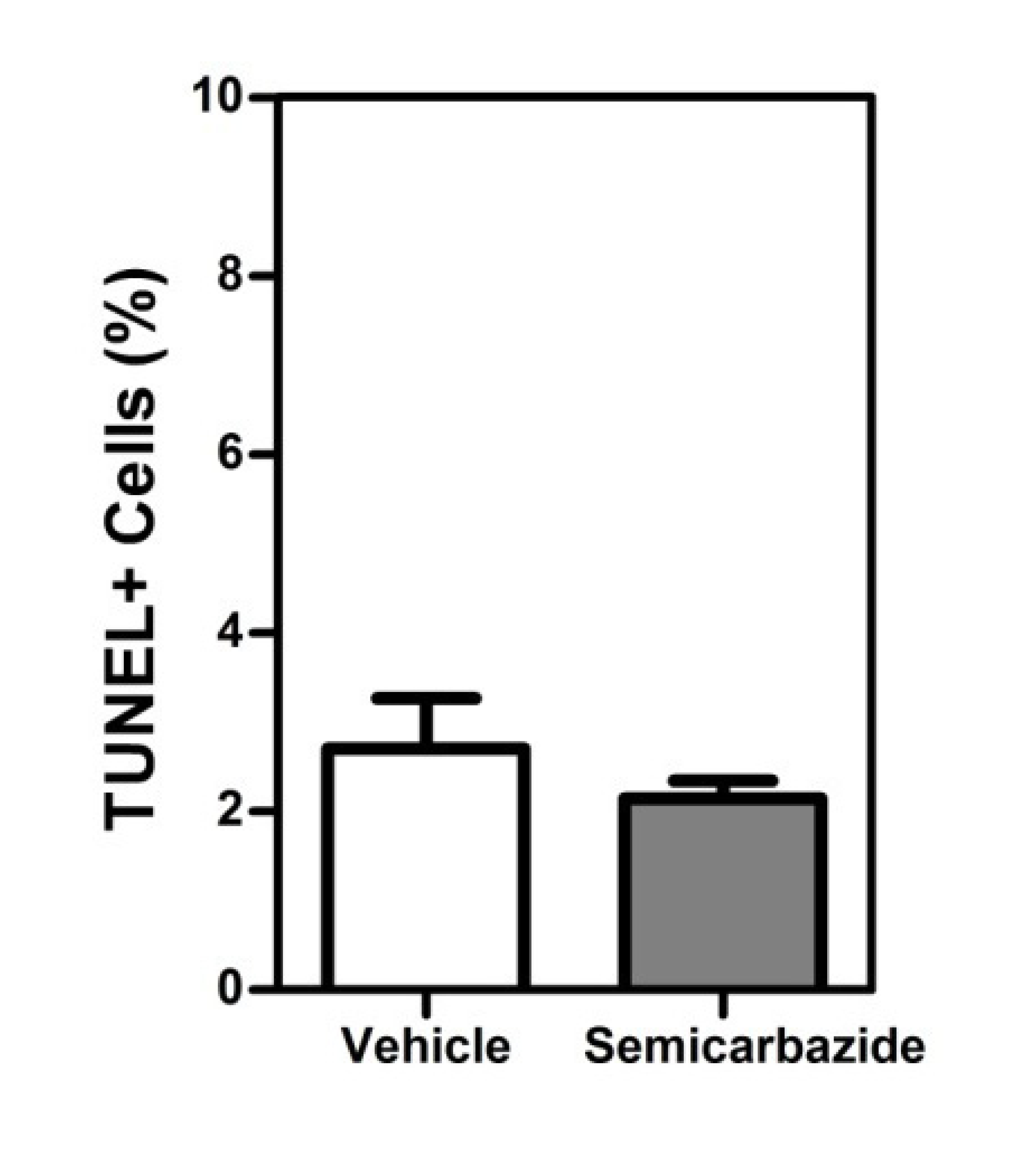

Supplement: S4 Fig — Female LDLr KO mice were treated as described in the legend to S1A Fig. TUNEL staining was perform to detect the apoptotic cells. Values represent the mean ±SEM. (TIF) [file pone.0152758.s004.tif]

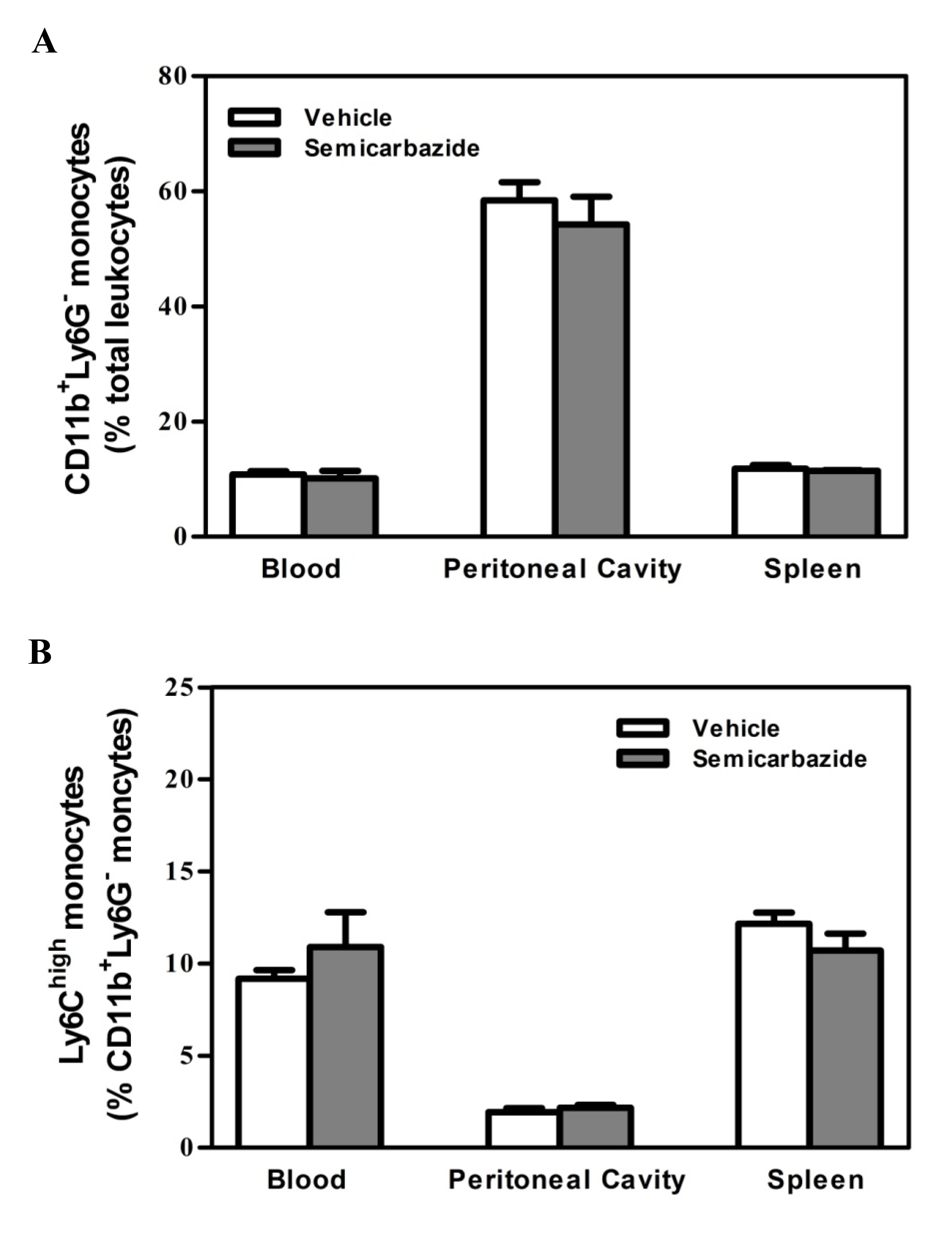

Supplement: S5 Fig — Male LDLr KO mice were treated as described in the legend to S1B Fig. Upon sacrifice, the percentage of CD11b+Ly6G- (A) and CD11b+Ly6G-Ly6Chigh monocytes (B) in the blood, peritoneal cavity, and spleen were analyzed by flow cytometry. Comparable absolute numbers of total cells were obtained in corresponding tissues of mice from each group. Results were expressed as mean±SEM. (TIF) [file pone.0152758.s005.tif]
